# Educating Patients by Providing Timely Information Using Smartphone and Tablet Apps: Systematic Review

**DOI:** 10.2196/17342

**Published:** 2020-04-13

**Authors:** Thomas Timmers, Loes Janssen, Rudolf B Kool, Jan AM Kremer

**Affiliations:** 1 IQ healthcare Radboud Institute for Health Sciences Radboud University Medical Center Nijmegen Netherlands; 2 Interactive Studios Rosmalen Netherlands; 3 Máxima Medical Center Veldhoven Netherlands

**Keywords:** patient education, push notification, self-management, eHealth, timely information, timely education, smartphone, tablet computer, self-care, mobile phone

## Abstract

**Background:**

Patient education is a crucial element within health care. It is a known predictor for increased engagement in shared decision making, improved medication and treatment adherence, higher levels of satisfaction, and even better treatment outcomes. Unfortunately, often patients only remember a very limited amount of medical information. An important reason is that most patients are simply not capable of processing large amounts of new medical information in a short time. Apps for smartphones and tablets have the potential to actively educate patients by providing them with timely information through the use of push notifications.

**Objective:**

The objective of this systematic review is to provide an overview of the effects of using smartphone and tablet apps to educate patients with timely education. Within this review, we focused on patients that receive their care in a hospital setting. We assessed the effects of the interventions on outcomes, such as patients’ knowledge about their illness and treatment, adherence to treatment instructions and to medication usage, and satisfaction with the care they received.

**Methods:**

A comprehensive search of MEDLINE (Medical Literature Analysis and Retrieval System Online), Embase, CINAHL (Cumulative Index to Nursing and Allied Health Literature), and Web of Science was conducted. Randomized controlled trials (RCTs) published between January 2015 and November 2019 were eligible for inclusion. Two reviewers independently searched and screened articles, assessed study quality and risk of bias, and extracted the data. Due to the heterogeneity of populations, interventions, and outcomes, a meta-analysis was not deemed appropriate. Instead, a narrative synthesis is presented.

**Results:**

A total of 21 RCTs with 4106 participants were included. Compared to usual care, overall effectiveness of the interventions was demonstrated in 69% of the outcomes. Effectiveness increased to 82% when the intervention had a duration shorter than one month and increased to 78% when the intervention provided at least one push notification per week. The interventions showed the highest effects on satisfaction with information, adherence to treatment instructions and to medication usage, clinical outcomes, and knowledge.

**Conclusions:**

This review demonstrates that educating patients with timely medical information through their smartphones or tablets improves their levels of knowledge, medication or treatment adherence, satisfaction, and clinical outcomes, as well as having a positive effect on health care economics. These effects are most pronounced in interventions with a short duration (ie, less than a month) and with a high frequency of messages to patients (ie, once per week or more). With the knowledge that patient education is a predictor for improved outcomes and the fact that patients have obvious difficulties processing large amounts of new medical information, we suggest incorporating the delivery of timely information through smartphone and tablet apps within current medical practices.

## Introduction

Patient education is a crucial element within health care. Health care professionals provide patients with information about the origins of complaints, treatment options, prognosis, how to prepare for treatment, or how to manage one’s health during the recovery phase. Health care professionals educate their patients because knowledge is a known predictor for increased engagement in shared decision making, improved medication and treatment adherence, higher levels of satisfaction, and better outcomes [1,2].

Unfortunately, patients often only remember a limited amount of the medical information they receive. Many different factors contribute to this. Some of these factors are related to the health care professional, such as using jargon or communicating in a passive way. Other factors are related to the patient, such as age, learning style, and stress [3]. Another important reason is the fact that most patients are simply not capable of processing large amounts of new medical information in a short amount of time [4].

During the last decade, smartphones, tablets, and apps have become commonplace in our society. These innovations offer many new opportunities within health care, such as optimizing the process of patient education. Apps, for example, allow patients to look at medical information as often as they like, at any place, and at any time. The information is comprehensive and different modes of information delivery and interaction are available. Furthermore, push notifications allow health care providers to actively educate patients with timely information, which, in this review, is defined as providing patients with small pieces of information at the time that these are actually relevant to them.

Although interventions like these appear to have much potential in allowing patients to better understand and to remember medical information, an overview of all available evidence on the effectiveness of these technologies has thus far not been published. The objective of this systematic review is to provide an overview of the effectiveness of educating patients by providing timely information using smartphone and tablet apps. With this systematic review, we focused on patients that receive care in a hospital setting rather than in primary care. We have chosen to do so since projects in primary care have already demonstrated effectiveness of electronic health (eHealth) apps, but these primarily focused on chronically ill patients from a population perspective and on telemonitoring services from an intervention perspective.

In this paper, we assess the effects that these interventions have on outcomes, such as patients’ knowledge about their illness and treatment options, adherence to medication or instructions, and satisfaction with the information or the care they received.

## Methods

### Search Strategy and Data Sources

To identify relevant studies, we used a two-step strategy. First, we conducted a preliminary search in PubMed to identify key articles, relevant keywords, and Medical Subject Headings (MeSH) terms. The second step was to have the search strategy be peer reviewed by an information specialist from the Radboud academic medical center’s medical library. Multimedia Appendix 1 shows the search strategy for the final search. We comprehensively searched the following databases: MEDLINE (Medical Literature Analysis and Retrieval System Online) (Ovid); Embase (Elsevier); CINAHL (Cumulative Index to Nursing and Allied Health Literature) (EBSCO); and Web of Science. Relevant systematic reviews were also assessed for eligible articles. In order to compare the effectiveness of interventions, we preferred to only include randomized controlled trials (RCTs). Since we were unsure about the number and quality of RCTs, our primary search also included cohort and quasi-experimental studies. After assessing the number and quality of RCTs, we decided to only include these in the review. Reporting was done in accordance with the PRISMA (Preferred Reporting Items for Systematic Reviews and Meta-Analyses) guidelines [5].

Based on the results of our preliminary search, we deliberately limited our search to articles published between January 1, 2015, and November 1, 2019, as the interventions described before this period did not meet the eligibility criteria or could no longer be repeated since the technique was outdated or no longer available. We searched for papers in English and looked at reference lists of included studies to optimize our search.

### Eligibility Criteria

RCTs were included if they met a number of eligibility criteria: (1) interventions had a focus on patient education through a smartphone or tablet app, used in a hospital setting; (2) interventions had to use push notifications to actively notify patients about newly available information in the app; and (3) the intervention had to be available for multiple days.

We excluded trials that focused solely on the acceptance or feasibility of technology, content or design of the intervention, availability in app stores, telemedicine (ie, remote care), websites or online platforms, or trials that only described the usage of an SMS. Furthermore, articles focusing on data collection, security, behavior or characteristics of patients, and health care professionals were excluded, as were study protocols. Studies were not excluded on the basis of sociodemographic characteristics of patients, such as age, gender, ethnicity, or any other related characteristic.

### Data Selection, Extraction, and Management

The search results from different electronic databases were combined within a single Endnote library, version 8.2 (Clarivate Analytics), and duplicates were removed. Two reviewers (TT and LJ) independently screened titles and abstracts to identify studies that potentially met the inclusion criteria. The full text of these articles was retrieved and read. Two review authors (TT and LJ) independently assessed these articles against the eligibility criteria and extracted the data from the included studies using a structured data extraction form. Disagreements were resolved through discussion and, if necessary, a third reviewer (RBK) was consulted. We extracted information about the patient population, outcomes, interventions, controls, results, and outcome measures.

### Assessment of Risk of Bias

Two reviewers (TT and LJ) independently assessed the risk of bias of included RCTs using the Cochrane Collaboration’s *risk of bias* tool [6]. Judgements concerning the risk of bias for each study were classified as high, some concerns, or low.

### Data Synthesis

Included studies were insufficiently homogenous in terms of patient population, outcomes, and type of intervention. The decision not to perform a meta-analysis was made as a consensus by all authors. For any outcome that was investigated in three or more studies, we present a narrative synthesis of results. In order to compare the effects of the different interventions over the different studies, a standardized mean difference (SMD) is reported, including the 95% CI for the effect. SMD is reported only when results are normally distributed and mean and SD are available. The magnitude of the effect is interpreted according to Cohen’s guidelines: small (SMD is 0.2 or lower), medium (SMD is between 0.2 and 0.8), or large (SMD is 0.8 or higher) [7].

Furthermore, we created a narrative synthesis of overall results per outcome in relation to the duration of the intervention or the frequency with which messages were sent to the patient. Therefore, the duration of the intervention was subdivided into short (<1 month) and long (≥1 month). The frequency of messaging was subdivided into high (>1 message per week) or low (≤1 message per week). The relative effectiveness was calculated by dividing the total number of participants in studies that demonstrated an effect for the outcome by the total number of participants in studies linked to the outcome. Finally, a weighted overall effect was calculated summarizing all outcomes, specified for the duration of the intervention and the frequency of messages.

## Results

### Overview

**Figure 1:**
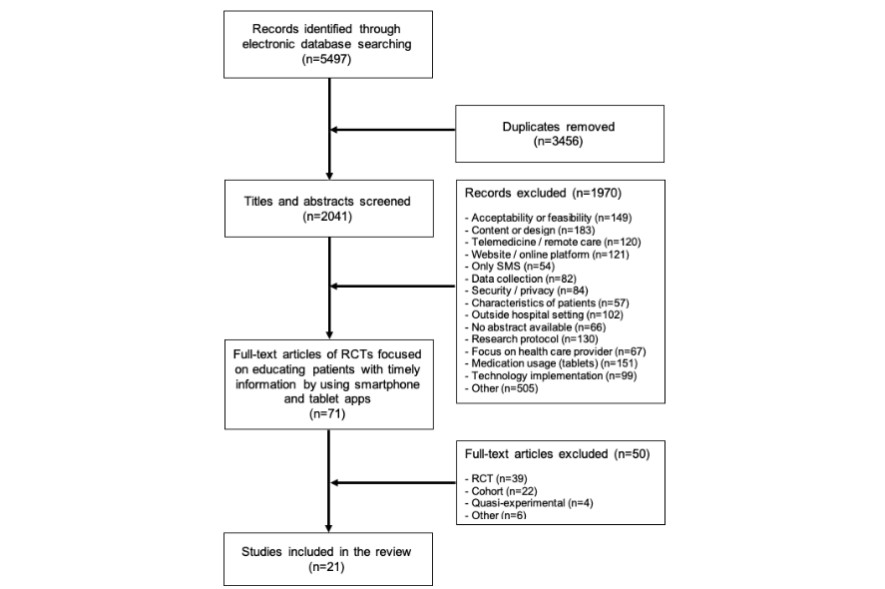
PRISMA (Preferred Reported Items for Systematic Reviews and Meta-Analyses) flowchart. RCT: randomized controlled trial.

Our searches yielded a total of 5497 articles from which 2041 unique articles were derived. After screening titles and abstracts, 1970 records were excluded. A total of 71 articles were assessed for eligibility by full-text screening. A total of 50 articles were excluded after full-text reading because of study type (ie, cohort, quasi-experimental, or other) or because the intervention used did not actually deliver timely education. In total, 21 RCTs were included in the review, including 4106 participants (see Figure 1). Sample sizes ranged from 34 participants [8] to 650 participants [9].

### Included Studies: Study Designs and Populations

Nine studies were conducted in Europe [10-18], four studies in North America [8,19-21], five studies in Asia [9,22-27], and one study in Africa [28]. In total, 4106 patients participated in the studies. Studies were divided over many different medical departments: gastroenterology [9,18,22,24,28], orthopedics [10,12,13], cardiology [17,20,25,26], oncology [21], surgery [11,19,23], urology [16], internal medicine [27], sports medicine [14], pulmonary disease [8], and neurology [15]. Six studies used a social media platform as the medium for the intervention [9,22-24,26,27]. Eight studies used apps that were already commercially available [10,12-14,16,17,19,20] and five studies used apps that were developed specifically for the study [8,18,21,25,28]. A total of five interventions that were used provided the possibility to interact with a health care provider [9,22,26-28].

Two studies included detailed information about the content and timing of notifications used in the intervention [10,17] and eight provided some details or images [9,12,13,15,21,22,25,28]. Regarding the phase of the treatment in which the study was conducted, seven studies focused on the period before the start of the treatment [9,12,18,21,22,24,28], 12 studies focused on the period after the start of the treatment [8,10,11,13-16,19,20,25-27], and one focused on both [23].

Details of the population, type of intervention, outcomes, and mean age of participants are presented in Table 1. The details of the interventions used, their duration, phase of the treatment, and frequency of notifying patients are presented in Table 2. An overview of all the measurement instruments used per study to assess these outcomes can be found in Multimedia Appendix 2.

**Table 1:** Details of the publications, interventions, outcomes, and populations.

Study	Year	Country	Department	Population (n)	Age (years), mean	Outcomes
Wang [22]	2019	China	Gastroenterology	Colonoscopy (392)	52	Bowel preparation adherence, quality of preparation, adenoma detection, and satisfaction
Timmers [10]	2019	Netherlands	Orthopedics	Knee replacement (212)	65	Pain, QoL^a^, physical functioning, satisfaction, and health care consumption
Mata [19]	2019	Canada	Surgery	Colorectal surgery (97)	60	Recovery protocol adherence, length of stay, complications, and satisfaction
Li [23]	2019	China	Surgery	Pediatric day-care surgery (127)	4^b^	Quality of recovery, satisfaction, and time consumption during follow-up
Jeon [24]	2019	South Korea	Gastroenterology	Colonoscopy (281)	48	Bowel preparation adherence, quality of preparation, and adenoma detection
Van der Meij [11]	2018	Netherlands	Surgery	Abdominal surgery (344)	52	Return to work, first return to normal activity, physical functioning, QoL, and satisfaction
Timmers [12]	2018	Netherlands	Orthopedics	Knee replacement (213)	62	Knowledge, mobile device proficiency, treatment chosen, and satisfaction
Najafi Ghezeljeh [26]	2018	Iran	Cardiology	Hypertension (100)	65	Hypertension self-management
Hardt [13]	2018	Germany	Orthopedics	Knee replacement (60)	65	Range of motion, pain, and physical functioning
Alanzi [27]	2018	Saudi Arabia	Internal medicine	Diabetes mellitus (92)	41^c^	Knowledge and self-efficacy
Widmer [20]	2017	United States	Cardiology	Cardiac rehabilitation (80)	64	In-person hospital visits, clinical values, QoL, and mood
Asklund [16]	2017	Sweden	Urology	Stress urinary incontinence (123)	45	Symptom severity and QoL
Sharara [28]	2017	Lebanon	Gastroenterology	Colonoscopy (160)	53	Bowel preparation adherence, quality of preparation, and satisfaction
Perry [8]	2017	United States	Pulmonary disease	Asthma (34)	15	Asthma control and expiratory volume
Lee [21]	2017	United States	Oncology	Breast cancer (120)	52	Knowledge, readiness for mammography, and satisfaction
Lakshminarayana [15]	2017	United Kingdom	Neurology	Parkinson disease (158)	60	Medication adherence, QoL, quality of consultation, anxiety and depression, and beliefs about medication
Guo [25]	2017	China	Cardiology	Atrial fibrillation (209)	68	Knowledge, QoL, adherence, and satisfaction
Van Reijnen [14]	2017	Netherlands	Sports medicine	Ankle trauma (220)	38	Incidence of ankle sprains, residual pain, and ankle disability
Kang [9]	2016	China	Gastroenterology	Colonoscopy (650)	45	Bowel preparation adherence and compliance with instructions
Johnston [17]	2016	Sweden	Cardiology	Myocardial infarction (174)	57	Medication adherence, satisfaction, and QoL
Lorenzo-Zuniga [18]	2015	Spain	Gastroenterology	Colonoscopy (260)	50	Bowel preparation adherence and satisfaction

^a^QoL: quality of life.

^b^Age of the children who underwent surgery. In the study, their parents (age not mentioned) used the app and provided the data.

^c^Study only reports that 75% of the participants were 41 years or older.

**Table 2:** Details and duration of the interventions used, frequency of notifying patients, and treatment phase.

Study	Year	Country	Intervention and control	Duration	Notification frequency	Treatment phase^a^
Wang [22]	2019	China	Dietary preparation through the WeChat platform in the days before colonoscopy, as well as timing and usage of the bowel preparation solution; possibility to ask questions as well Control: Standard written information	3 days	Daily	Pre
Timmers [10]	2019	Netherlands	Day-to-day information and videos through an app on pain, wound care, physiotherapy exercises, medication usage, and self-care in the early postoperative phase after total knee replacement Control: Simplified version of the app with only basic information	28 days	Daily	Post
Mata [19]	2019	Canada	Recovery targets and educational information through an app on how to achieve them in the first days after surgery Control: Standard written instructions	2-4 days	Daily	Post
Li [23]	2019	China	Recovery education through the WeChat platform in the days before and after surgery Control: Telephone call by nursing staff	2-4 days	Daily	Pre/post
Jeon [24]	2019	South Korea	Self-management education through the WeChat platform in the days before colonoscopy by using videos Control: Standard written information	3 days	Daily	Pre
Van der Meij [11]	2018	Netherlands	Personalized eHealth^b^ program through an app for patients undergoing abdominal surgery Control: Placebo website with standard recovery advice	3 months	Weekly	Post
Timmers [12]	2018	Netherlands	Subdivided and interactive information through an app in the week prior to the consultation with an orthopedic surgeon because of possible knee osteoarthritis Control: Standard information on website	7 days	Daily	Pre
Najafi Ghezeljeh [26]	2018	Iran	Self-management education through the Telegram platform in the weeks after hospitalization Control: Standard written information	6 weeks	Weekly	Post
Hardt [13]	2018	Germany	Postoperatively app-based, feedback-controlled, active muscle training program Control: Standard physiotherapy sessions	4 days	Daily	Post
Alanzi [27]	2018	Saudi Arabia	Diabetes mellitus education through the WhatsApp platform (eg, signs and symptoms, diet, and exercises) Control: Standard written information	8 weeks	Weekly	Post
Widmer [20]	2017	United States	Reporting of dietary and exercise habits through an app, as well as educational information on lifestyle during cardiac rehabilitation Control: Web-based platform	3 months	Occasionally	Post
Asklund [16]	2017	Sweden	Treatment program for pelvic floor muscles and information about stress urinary incontinence and lifestyle through an app Control: Standard written instructions	3 months	Daily	Post
Sharara [28]	2017	Lebanon	Dietary preparation through an app in the days before colonoscopy, as well as timing and usage of the bowel preparation solution Control: Standard written instructions	4 days	Daily	Pre
Perry [8]	2017	United States	Education on medication usage and peak flow or asthma logging through an app Control: Standard written instructions	6 months	Occasionally	Post
Lee [21]	2017	United States	Personal, tailored multimedia messages through an app to prepare women for breast cancer screening Control: Standard written instructions	7 days	Daily	Pre
Lakshminarayana [15]	2017	United Kingdom	Reminding patients about medication usage, tracking of self-management skills, and educating patients about Parkinson disease through an app Control: Standard written instructions	4 months	Occasionally	Post
Guo [25]	2017	China	Educational program about atrial fibrillation and how to self-manage at home Control: Standard written instructions	3 months	Occasionally	Post
Van Reijnen [14]	2017	Netherlands	Neuromuscular training program through an app for athletes who suffered a sprained ankle Control: Standard written instructions	2 months	Occasionally	Post
Kang [9]	2016	China	Dietary preparation through the WeChat platform in the days before colonoscopy, as well as timing and usage of the bowel preparation solution; possibility to ask questions as well Control: Standard written instructions	4 days	Daily	Pre
Johnston [17]	2016	Sweden	Educational messages based on the data patients had registered about their medication usage Control: Simplified version of the app with only basic information	6 months	Weekly	Post
Lorenzo-Zuniga [18]	2015	Spain	Dietary preparation through an app in the days before colonoscopy, as well as timing and usage of the bowel preparation solution Control: Standard written instructions	4 days	Daily	Pre

^a^Pre: before the start of the treatment; post: after the start of the treatment.

^b^eHealth: electronic health.

### Risk of Bias of Included Studies

**Figure 2:**
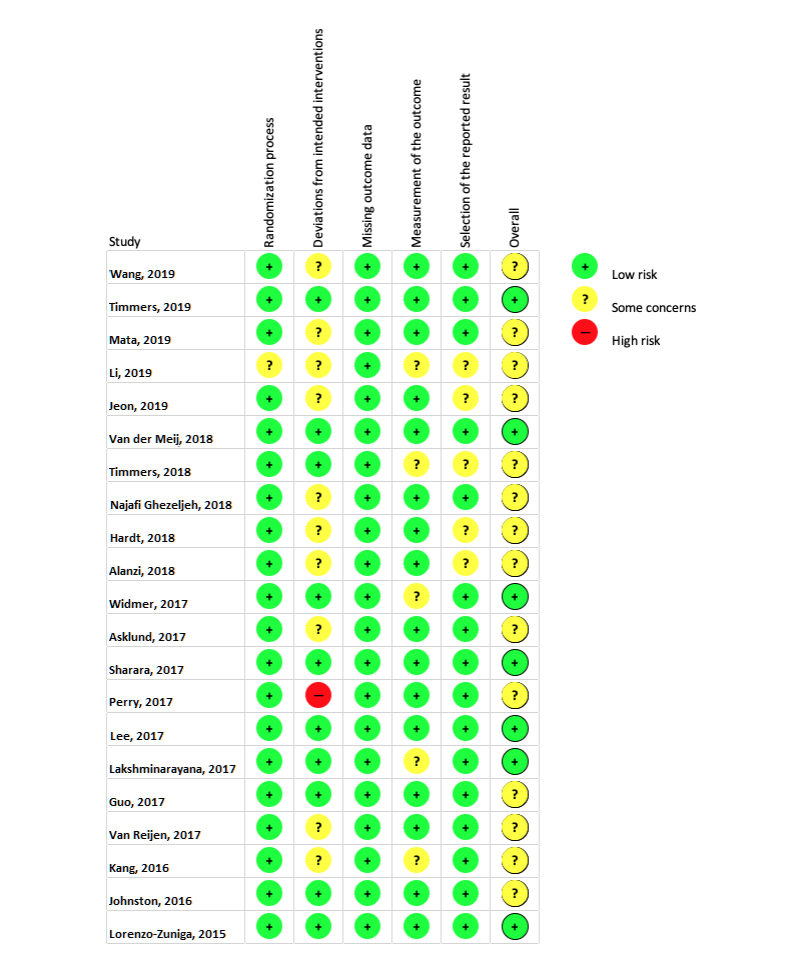
Level of risk of bias, per study, per domain.

**Figure 3:**
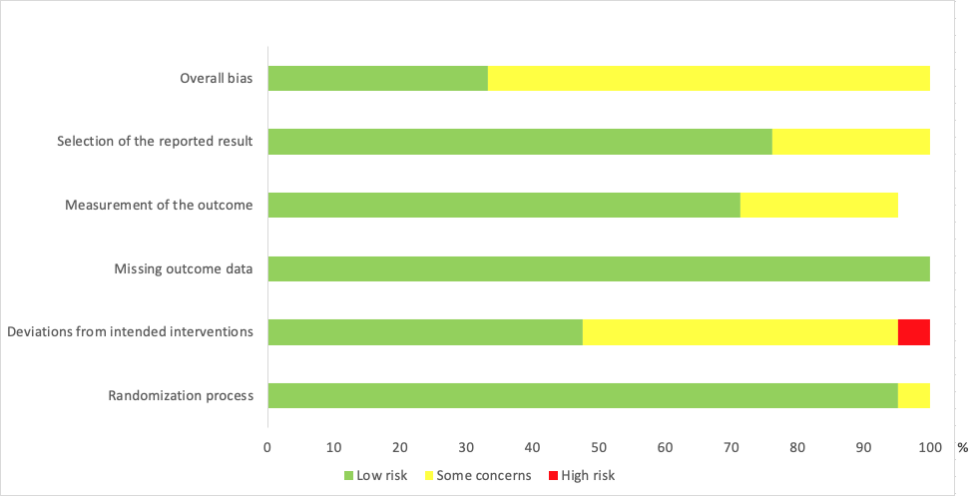
Risk of bias presented as the percentage of studies with low risk, some concerns, or high risk, scored for the different domains of bias.

All 21 included studies were assessed for risk of bias in the following domains: selection of the reported result, measurement of the outcome, missing outcome data, deviations from intended interventions, and randomization process. The levels of risk—low, some concerns, or high—per study, per domain are presented in Figure 2. An overview of the percentage of studies related to the level of risk and domain of bias is presented in Figure 3.

### Outcomes

#### Overview

Characteristics of the included studies are presented per outcome. Per study, the effect of the intervention on the outcome is described as *in favor of the intervention group*, *in favor of the control group*, or *no effect*.

#### Satisfaction

A total of 12 RCTs [10-12,15,17-19,21,22,25,28], in which 2466 patients participated, reported results related to satisfaction. Two main themes emerged from these studies: satisfaction with the information provided [10-12,17,19,21,28] and satisfaction with the overall care that was delivered [10-12,18,22,25] (see Table 3).

Regarding patients’ satisfaction with the information, an effect in favor of the intervention group was demonstrated in eight out of 10 studies. Interventions included an app that was used to educate patients about the preparation for their colonoscopy [28], consultation with an orthopedic surgeon [12], postoperative self-management after knee replacement surgery [10], breast cancer screening [21], healthy lifestyle interventions in myocardial infarction patients [17], and return to normal activities after abdominal surgery [11]. One study, which focused on enhanced recovery education after colorectal surgery [19], showed no difference in terms of satisfaction between the intervention and control groups. SMD ranged from medium to large in five studies [10-12,17,21] and could not be calculated for the two other studies.

Regarding patients’ satisfaction with the overall care they received, an effect in favor of the intervention group was demonstrated in four out of eight studies. These studies measured the patient-perceived level of involvement by the hospital after discharge [10], satisfactory bowel preparation [22], satisfaction with anticoagulation therapy [25], level of patient-centered care in Parkinson disease [15], and overall experience with the bowel preparation process [18]. Three other studies showed no differences between groups in patients’ overall satisfaction with care related to abdominal surgery [11], patients’ satisfaction related to the consultation with their orthopedic surgeon [12], and patients’ overall satisfaction with the recovery process after pediatric surgery [23]. SMD ranged from small to large in six studies [10-12,15,18,22,25] and could not be calculated for the other study.

**Table 3:** Details about patients’ satisfaction.

Satisfaction type and study		Population (n)	Description^a^	Effect^b^	SMD^c^ (95% CI)
**Satisfaction with information provided**					
	Van der Meij [11]	Abdominal surgery (344)	Personalized information on activity resumption	+	0.43 (0.22 to 0.65)
	Lee [21]	Breast cancer (120)	Breast cancer screening instructions	+	0.55 (0.19 to 0.90)
	Sharara [28]	Colonoscopy (160)	Bowel preparation	+	SMD could not be calculated^d^
	Mata [19]	Colorectal surgery (97)	Postoperative adherence protocol	=	SMD could not be calculated^e^
	Timmers [10]	Knee replacement (212)	Education on pain management, exercises, and self-care	+	0.97 (0.68 to 1.27)
	Timmers [12]	Knee replacement (213)	Level of knowledge about treatment options	+	0.54 (0.26 to 0.82)
	Timmers [12]	Knee replacement (213)	Preparation for medical consultation	+	0.70 (0.42 to 0.98)
	Johnston [17]	Myocardial infarction (174)	Overall satisfaction with the app	+	0.56 (0.23 to 0.88)
**Satisfaction with care received**					
	Van der Meij [11]	Abdominal surgery (344)	Overall satisfaction with care received	=	0.20 (–0.01 to 0.41)
	Guo [25]	Atrial fibrillation (209)	Overall satisfaction with care received	+	0.58 (0.15 to 1.00)
	Wang [22]	Colonoscopy (392)	Colonoscopy treatment itself	+	SMD could not be calculated^f^
	Lorenzo-Zuniga [18]	Colonoscopy (260)	Overall satisfaction with care received	+	0.78 (0.52 to 1.04)
	Timmers [10]	Knee replacement (212)	Hospital involvement during recovery	+	0.89 (0.60 to 1.19)
	Timmers [12]	Knee replacement (213)	Medical consultation with orthopedic surgeon	=	0.29 (–0.02 to 0.58)
	Lakshminarayana [15]	Parkinson disease (158)	Overall satisfaction with care received (Patient-Centered Outcomes Questionnaire for Parkinson’s Disease)	+	0.35 (0.03 to 0.67)
	Li [23]	Surgery (127)	Overall quality of recovery	=	0.20 (–0.15 to 0.55)

^a^All items were patient reported versus clinician reported.

^b^Effects were in favor of the intervention group (+) or there were no effects (=). No study had effects in favor of the control group (–).

^c^SMD: standardized mean difference.

^d^Outcome only measured in intervention group.

^e^No SD available (only average and *P* value).

^f^Nonnormal distributed data.

#### Adherence

A total of 11 RCTs [9,15,17-19,22,24-28], in which 2573 patients participated, reported results related to adherence. Two main themes emerged from these studies: adherence to treatment instructions [9,18,19,22,24,26-28] and adherence to medication usage [15,17,25] (see Table 4).

Regarding patients’ adherence to treatment instructions, an effect in favor of the intervention group was demonstrated in five out of eight studies, focusing on patients’ self-management in diabetes mellitus [27], hypertension [26], and adherence to purgative and dietary instructions for bowel preparation before their colonoscopy [9,22,24]. No differences between groups were reported in two other studies focusing on preparation for colonoscopy [18,28] and a postoperative recovery program after colorectal surgery [19]. SMD ranged from small to large in six studies [9,18,24,26-28], was negative in one study [19], and could not be calculated for the other study.

Regarding patients’ adherence to their medication usage, an effect in favor of the intervention group was demonstrated in all three studies addressing this theme. These studies focused on drug adherence in Parkinson disease [15], anticoagulation adherence in patients who suffered from atrial fibrillation [25], or myocardial infarction [17]. With regard to the latter, patients in the intervention group reported lower missed medication doses. However, the same study also reported that there were no differences between groups in results related to the medication adherence questionnaire that was assessed. SMD ranged from small to medium in two studies [15,17] and could not be calculated for the other study.

**Table 4:** Details about patients’ adherence.

Adherence type and study		Population (n)	Description^a^	Effect^b^	SMD^c^ (95% CI)
**Adherence to instructions**					
	Wang [22]	Colonoscopy (392)	Purgative and dietary instructions for bowel preparation (CR)	+	SMD could not be calculated^d^
	Jeon [24]	Colonoscopy (281)	Purgative and dietary instructions for bowel preparation (PR)	+	SMD could not be calculated^e^
	Jeon [24]	Colonoscopy (281)	Clinical Bowel Preparation score (CR)	+	0.28 (0.05 to 0.52)
	Sharara [28]	Colonoscopy (160)	Purgative and dietary instructions for bowel preparation (PR)	=	SMD could not be calculated^d^
	Sharara [28]	Colonoscopy (160)	Clinical Bowel Preparation score (CR)	=	0.12 (–0.19 to 0.43)
	Kang [9]	Colonoscopy (650)	Purgative and dietary instructions for bowel preparation (CR)	+	0.51 (0.37 to 0.66)
	Lorenzo-Zuniga [18]	Colonoscopy (260)	Purgative and dietary instructions for bowel preparation (CR)	=	0.16 (–0.08 to 0.42)
	Mata [19]	Colorectal surgery (97)	Postoperative recovery elements (eg, mobilization) (PR)	=	–0.13 (–0.52 to 0.26)
	Alanzi [27]	Diabetes mellitus (92)	Self-efficacy in diabetes mellitus	+	0.78 (0.36 to 1.21)
	Najafi Ghezeljeh [26]	Hypertension (100)	Hypertension self-management (PR)	+	6.78 (5.34 to 8.21)
**Adherence to medication**					
	Lakshminarayana [15]	Parkinson disease (158)	Parkinson disease drug adherence (PR)	+	0.37 (0.05 to 0.68)
	Guo [25]	Atrial fibrillation (209)	Anticoagulation drug adherence (PR)	+	SMD could not be calculated^e^
	Johnston [17]	Myocardial infarction (174)	Anticoagulation drug adherence (PR)	=	SMD could not be calculated^d^
	Johnston [17]	Myocardial infarction (174)	Missed medication doses (PR)	+	0.14 (–0.16 to 0.46)

^a^Items were either clinician reported (CR) or patient reported (PR).

^b^Effects were in favor of the intervention group (+) or there were no effects (=). No study had effects in favor of the control group (–).

^c^SMD: standardized mean difference.

^d^No SD available (only average and *P* value).

^e^Nonnormal distributed data.

#### Quality of Life

Seven RCTs [10,11,15-17,20,25], in which 1300 patients participated, reported results related to quality of life (see Table 5). An effect in favor of the intervention group was demonstrated in four studies. These studies measured the effect of the intervention on quality of life at four weeks after knee replacement surgery [10], three months after starting a program for cardiac rehabilitation [20], three months after starting a program for pelvic floor muscle training [16], and three months after starting a program for enhanced self-management after atrial fibrillation [25]. Three studies did not report an effect in the intervention group at the following time points: 6 months after intermediate-grade abdominal surgery [11], 4 months after starting a self-management program in Parkinson disease [15], and 6 weeks after starting a support program on lifestyle changes and drug adherence in myocardial infarction patients [17]. SMD ranged from small to large in five studies [10,15,20] and could not be calculated for two studies [11,25].

**Table 5:** Details about patients’ quality of life.

Study	Population (n)	Description^a^	Effect^b^	SMD^c^ (95% CI)
Van der Meij [11]	Abdominal surgery (344)	After abdominal surgery	=	SMD could not be calculated^d^
Guo [25]	Atrial fibrillation (209)	After starting atrial fibrillation management program	+	SMD could not be calculated^d^
Widmer [20]	Cardiac rehabilitation (80)	After starting cardiac rehabilitation	+	3.30 (2.60 to 4.02)
Timmers [10]	Knee replacement (212)	After knee replacement surgery	+	0.44 (0.15 to 0.72)
Johnston [17]	Myocardial infarction (174)	After starting lifestyle and drug adherence support	=	0.33 (0.01 to 0.66)
Lakshminarayana [15]	Parkinson disease (158)	After starting self-management app	=	0.18 (–0.14 to 0.49)
Asklund [16]	Stress urinary incontinence (123)	After starting pelvic floor muscle training	+	0.81 (0.44 to 1.18)

^a^All items were patient reported versus clinician reported.

^b^Effects were in favor of the intervention group (+) or there were no effects (=). No study had effects in favor of the control group (–).

^c^SMD: standardized mean difference.

^d^No SD available (only average and *P* value).

#### Clinical Outcomes

A total of 11 RCTs [8,10,11,13-16,20,22,24,28], in which 1783 patients participated, reported results related to clinical outcomes. Three main themes emerged from these studies: physical functioning and pain [10,11,13,14], clinical values [20,22,24,28], and symptoms [8,15,16] (see Table 6).

Regarding physical functioning, an effect in favor of the intervention group was demonstrated in three out of four studies, albeit not on all outcomes. These results were related to physical functioning after abdominal surgery [11] and pain and knee function after knee replacement surgery [10,13]. No differences between groups were reported concerning pain and activities after abdominal surgery [11] or concerning knee function and physiotherapy assessment tests [13]. One study related to ankle function after sports-related trauma did not demonstrate a difference between groups either [14]. SMD was medium in one study [10] and could not be calculated for the other studies.

Regarding clinical values, an effect in favor of the intervention group was demonstrated in at least one of the outcomes of all four included studies. These effects were related to weight loss during cardiac rehabilitation [20] and adenoma detection during colonoscopy [22,24,28]. No differences between groups were found concerning cholesterol, glucose, and exercise capacity in cardiac rehabilitation [20]. SMD ranged from small to large in two studies [15,16] and could not be calculated for the other study.

Regarding symptoms, an effect in favor of the intervention group was demonstrated in one out of three studies. These results were related to a decrease in symptom severity after using an intervention to train pelvic floor muscles in women who suffer from stress-related urinary incontinence [16]. No differences between groups were reported in nonmotor symptoms related to Parkinson disease [15] and asthma [8]. SMD ranged from small to large within one study [20] and could not be calculated for the other studies.

**Table 6:** Details about clinical parameters.

Clinical parameters and study, population, and description^a^				Effect^b^	SMD^c^ (95% CI)
**Physical functioning and pain**					
	**Van der Meij [11]**				
		**Abdominal surgery (n=344)**			
			Physical function (PR)	+	SMD could not be calculated^d^
			Physical activities (PR)	=	SMD could not be calculated^d^
			Recovery (PR)	=	SMD could not be calculated^d^
			Pain intensity (PR)	=	SMD could not be calculated^d^
	**Van Reijnen [14]**				
		**Ankle trauma (n=220)**			
			Ankle function (PR)	=	SMD could not be calculated^e^
	**Hardt [13]**				
		**Knee replacement (60)**			
			Knee range of motion (CR)	+	SMD could not be calculated^e^
			Pain at rest (PR)	=	SMD could not be calculated^e^
			Pain in motion (PR)	+	SMD could not be calculated^e^
			Knee function (PR)	=	SMD could not be calculated^e^
			Assessment tests (CR)	=	SMD could not be calculated^e^
	**Timmers [10]**				
		**Knee replacement (n=212)**			
			Pain at rest (PR)	+	0.51 (0.23 to 0.79)
			Pain during activity (PR)	+	0.49 (0.21 to 0.77)
			Pain during the night (PR)	+	0.42 (0.14 to 0.71)
			Knee function (PR)	+	0.47 (0.19 to 0.76)
**Clinical values**					
	**Widmer [20]**				
		**Cardiac rehabilitation (n=80)**			
			Weight (CR)	+	0.80 (0.32 to 1.28)
			Cholesterol (CR)	=	0.49 (–0.07 to 0.87)
			Glucose (CR)	=	0.05 (–0.41 to 0.52)
			Rehabilitation session attended (CR)	=	0.28 (–0.19 to 0.74)
			Exercise capacity (VO_2_ peak) (CR)	=	0.22 (–0.24 to 0.69)
	**Wang [22]**				
		**Colonoscopy (n=392)**			
			Adenoma detection rate (1 adenoma detected) (CR)	=	SMD could not be calculated^d^
			Adenoma detection rate (>1 adenoma detected) (CR)	+	SMD could not be calculated^d^
	**Jeon [24]**				
		**Colonoscopy (n=281)**			
			Adenoma detection rate (overall) (CR)	+	SMD could not be calculated^e^
	**Sharara [28]**				
		**Colonoscopy (n=160)**			
			Adenoma detection rate (overall) (CR)	+	SMD could not be calculated^d^
**Symptoms**					
	**Perry [8]**				
		**Asthma (n=34)**			
			Asthma control rest (PR)	=	SMD could not be calculated^e^
	**Lakshminarayana [15]**				
		**Parkinson disease (n=158)**			
			Range of nonmotor symptoms (PR)	=	0.16 (–0.16 to 0.48)
	**Asklund [16]**				
		**Stress urinary incontinence (n=123)**			
			Symptom severity (PR)	+	0.95 (0.58 to 1.33)

^a^Items were either patient reported (PR) or clinician reported (CR).

^b^Effects were in favor of the intervention group (+) or there were no effects (=). No study had effects in favor of the control group (–).

^c^SMD: standardized mean difference.

^d^No SD available (only average and *P* value).

^e^Nonnormal distributed data.

#### Health Care Economics

Five RCTs [10,11,19,23], in which 860 patients participated, reported results related to health care economics (see Table 7). An effect in favor of the intervention group was demonstrated in three studies, concerning patients’ contact with health care providers after total knee replacement surgery [10] and after pediatric day-care surgery [23], as well as after returning to work after abdominal surgery [11]. The other studies did not report an effect in favor of the intervention group for patients undergoing colorectal or abdominal surgery [11,19] or patients attending a cardiac rehabilitation program [20]. Regarding 30-day hospital readmissions, an effect in favor of the control group was demonstrated after colorectal surgery [19]. SMD ranged from small to large in two studies [19,23] and could not be calculated for the other studies.

**Table 7:** Details of health care economics of studies.

Study, Population (n), Description^a^			Effect^b^		SMD^c^ (95% CI)	
**Van der Meij [11]**						
	**Abdominal surgery (344)**					
		Postoperative complications (CR)		=		SMD could not be calculated^d^
		Mean cost differences (CR)		=		SMD could not be calculated^d^
		Return to work (PR)		+		SMD could not be calculated^d^
		Return to 75% of normal activities (PR)		=		SMD could not be calculated^d^
**Widmer [20]**						
	**Cardiac rehabilitation (80)**					
		Emergency department visits (CR)		=		SMD could not be calculated^d^
		Rehospitalization (CR)		=		SMD could not be calculated^d^
		Emergency department visits plus rehospitalization (CR)		=		SMD could not be calculated^d^
**Mata [19]**						
	**Colorectal surgery (97)**					
		Length of stay (CR)		=		0.19 (–0.21 to 0.59)
		Postoperative complications (CR)		=		SMD could not be calculated^d^
		30-day reoperation (CR)		=		SMD could not be calculated^d^
		30-day emergency department visits (CR)		=		SMD could not be calculated^d^
		30-day hospital readmissions (CR)		–		SMD could not be calculated^d^
**Timmers [10]**						
	**Knee replacement (212)**					
		Contact with hospital, general practitioner, or home care organization during the 4 weeks after discharge (PR)		+		SMD could not be calculated^d^
**Li [23]**						
	**Surgery (127)**					
		Time consumed during follow-up (CR)		+		3.58 (3.02 to 4.14)

^a^Items were either clinician reported (CR) or patient reported (PR).

^b^Effects were in favor of the intervention group (+), in favor of the control group (–), or there were no effects (=).

^c^SMD: standardized mean difference.

^d^Nonnormal distributed data.

#### Knowledge

Four RCTs [10,21,25,27], in which 634 patients participated, reported results related to condition- or treatment-specific knowledge acquisition (see Table 8). An effect in favor of the intervention group was demonstrated in all four studies. All studies focused on disseminating disease-specific information, ranging from treatment options for patients with knee complaints due to osteoarthritis [12] to self-management in atrial fibrillation patients [25] or diabetes mellitus [27] and general knowledge about breast cancer and screening options [21]. SMD ranged from medium to large in three studies [12,21,27] and could not be calculated for one study.

**Table 8:** Details about disease-specific knowledge acquisition.

Study	Population (n)	Description^a^	Effect^b^	SMD^c^ (95% CI)
Guo [25]	Atrial fibrillation (209)	Knowledge about atrial fibrillation	+	SMD could not be calculated^d^
Lee [21]	Breast cancer (120)	Knowledge about breast cancer and screening options	+	0.32 (–0.04 to 0.68)
Alanzi [27]	Diabetes mellitus (92)	Knowledge about diabetes mellitus and lifestyle	+	4.65 (3.87 to 5.44)
Timmers [12]	Knee replacement (213)	Actual knowledge about treatment options	+	1.27 (0.95 to 1.60)
Timmers [12]	Knee replacement (213)	Perceived knowledge about treatment options	+	0.87 (0.56 to 1.18)

^a^All items were patient reported versus clinician reported.

^b^Effects were in favor of the intervention group (+) for all studies, versus effects in favor of the control group (–) or no effects (=).

^c^SMD: standardized mean difference.

^d^Nonnormal distributed data.

#### Narrative Synthesis of Overall Results

Overall results demonstrate an average effectiveness of the intervention of 69% (see Table 9). Satisfaction with information, adherence to instructions and medication, clinical outcomes (eg, weight loss or adenoma detection), and knowledge acquisition showed the highest effects (>70%). When taking into account the duration of the intervention, a clear advantage in terms of effect is demonstrated by the interventions that have a duration of less than one month, compared to the interventions that take more than one month: 82% effectiveness versus 69%. A clear difference is noted in the comparison between the frequencies of messaging patients with information as well: an average effectiveness of 78% in the high-frequency group (more than once per week, on average) versus 64% in the low-frequency group (once per week, on average).

**Table 9:** Synthesis of results: average effectiveness per outcome.

Outcome	Dimension	Number of studies/ population members	Average effectiveness^a^, %	Duration, %		Frequency, %		
				Short (<1 month)	Long (≥1 month)		High^b^	Low^c^
Satisfaction	Information provided	7/1320	93	88	100		88	100
Satisfaction	Overall care	8/1915	64	72	52		72	52
Adherence	Instructions	8/2032	75	72	100		75	N/A^d^
Adherence	Medication usage	3/541	84	N/A	84		50	100
Quality of life	Overall	7/1300	48	100	38		66	57
Clinical parameters	Physical functioning and pain	4/836	50	89	30		89	30
Clinical parameters	Clinical values	4/913	74	76	50		76	50
Clinical parameters	Symptoms	3/315	39	N/A	39		100	0
Health care economics	Overall	5/860	59	78	68		78	68
Knowledge	Overall	4/634	100	100	100		100	100
Average effect	N/A	N/A	69	82	69		78	64

^a^Average effectiveness is the weighted average of the population linked to an outcome and the part of the population with a positive effect on the outcome.

^b^High frequency is >1 message per week, on average.

^c^Low frequency is ≤1 message per week, on average.

^d^N/A: not applicable.

## Discussion

### Principal Findings

The objective of this systematic review was to evaluate the effectiveness of educating patients by providing timely information using smartphone and tablet apps. In particular, we focused on patients that had undergone treatment in a hospital. A total of 21 studies were identified, most with some concerns in terms of risk of bias. Included studies showed low levels of homogeneity in terms of populations and outcomes. Overall results demonstrate an average effectiveness of the interventions in 69% of the studies. Satisfaction with information, adherence to instructions and medication, improved clinical values (eg, weight loss or adenoma detection), and knowledge acquisition showed the highest effects (>70%). An overall effect of 82% was observed in studies that lasted less than one month. Studies with a higher frequency of messaging (ie, more than once per week) were associated with an average effect of 78%. These results should not only be considered effective from a single outcome point-of-view, but should be, from a more holistic perspective, considered as important components required for effective patient self-management support as well [29].

Our results are in line with earlier reviews that focus on the effect of eHealth interventions on multiple outcomes in chronic health conditions [30,31]. A review by Schoeppe et al reported a positive effect in terms of prevention by focusing on lifestyle changes, such as diet, exercise, and sedentary behavior [32]. The average duration of the interventions in the Schoeppe et al review was 8 weeks, which is longer than the average duration of interventions in our review. However, this is probably due to the fact that the interventions in the Schoeppe et al review focused on behavioral changes related to lifestyle, whereas studies in our review sometimes lasted only 3 or 4 days, in which the aim is not to change one’s lifestyle, but to optimize one’s preparation for a one-time event such as a colonoscopy. The usage of frequent notifications has been recognized to encourage greater exposure to the intervention’s content without deterring engagement [33].

Even though results seem to indicate that interventions of a short duration with a high frequency of notifications are beneficial to the patient, the low level of homogeneity across these studies makes it impossible to extract an optimal structure, duration, or frequency for messaging patients. Such a challenge has also been reported in a 2018 review on education via strategies and structures [34]. Unfortunately, only a few studies reported detailed information about the content that was provided to patients, its format (eg, text, photo, or video), and the actual timing of the content delivery. This information could have provided additional insights on what makes interventions successful or not.

Our results demonstrate the emerging character of this field of research: the 21 included studies were conducted in 10 different medical departments, covering 15 different types of treatments. Four medical specialties—cardiology, orthopedics, surgery, and gastroenterology—have had more than three studies included. Only interventions related to colonoscopy and knee replacement were studied more than once. The results regarding the number of studies that we excluded from this review also demonstrate that many studies still focus on feasibility, acceptance of technology, and the design and content of apps, rather than on the actual effect of this type of intervention.

### Strengths and Limitations

To our knowledge, this review is the first to assess the effectiveness of educating patients in preparation of, during, or after their treatment in the hospital using an app for smartphones or tablets. This review adopted a detailed and comprehensive search strategy, followed by robust screening, data extraction, and risk-of-bias assessment, adhering to the PRISMA guidelines. A total of 21 studies were found eligible for inclusion, seven of them having a *low risk* level of bias and 14 of them having a level of bias with *some concerns* according to Cochrane’s *risk of bias* assessment. The relatively large sample sizes allowed us to calculate SMDs and therewith enabled us to compare study outcomes. The observed high level of heterogeneity in terms of outcomes, population, and intervention characteristics, such as interaction models, commercial and noncommercial products, or social media platforms, made it inappropriate to perform a meta-analysis for any outcome.

In this review, we focused on the timely delivery of educational information to overcome patient-perceived information overload. The duration of the interventions within these studies ranged from 3 days to 6 months. In our opinion, this range is another indicator that this type of research is still at an early stage, in which the focus of the trial is really on the intervention itself instead of its long-term effects.

### Implications for Practice

The results of our review demonstrate the effective application of smartphone and tablet apps to educate patients with timely information. The effects are visible within various outcomes and across various medical specialties. Medical practices could benefit from these effects by combining two already-existing resources: patient education materials and smartphones and tablets. Patient education is already available on hospital websites, brochures, and through the oral advice of health care professionals. Additionally, more and more patients, as well as their surrounding caretakers, possess a smartphone or tablet. By adding the concept of *timing* to existing educational materials, one could improve the likelihood that patients receive the right information at the right time. By using the push notification mechanism on smart devices, patients can also be actively made aware of newly available information related to their treatment. Medical practices may choose to either build an app themselves or use already-available commercial products or platforms, social media or otherwise. After the initial development of an app, little or no further adjustments to existing workflows are needed for successful implementation, which is regarded as a crucial factor for successful eHealth implementation [35]. Of course, some patients may require support during the initial downloading or configuring of the app, but when this effort is compared to the possible benefits in terms of improved outcomes, satisfaction, and health care consumption as described in this review, these efforts appear worthwhile.

### Future Research

Delivering timely education to patients through an app for smartphones or tablets has the potential to contribute to the emerging field of patient education research, which may lead to a positive effect on numerous outcomes. Given the novelty of this area of research, more studies need to be performed in order to demonstrate the generalizability of the concept, as well as its long-term effects. In this review, we chose to include only RCTs, since this study design is currently considered to be the gold standard research design to assess the effectiveness of interventions. Yet, we believe it is legitimate to question whether this is the only appropriate study design, as eHealth innovations and research projects could be characterized by what we would like to refer to as “moving objects” and “moving targets.” By moving objects, we refer to the interventions themselves, as these may easily be adapted to the real-time needs of patients and health care providers by their inventors. By moving targets, we refer to outcomes that might not have been defined in the original research protocol but arose from the data and insights that were gathered during the study. Changing the intervention itself or adding outcomes during the course of a study is, however, often considered *not done*, as it could quickly lead to a high risk of bias and a lower overall quality of the research. As a consequence, many interventions might not be studied at all, because from a supplier’s or producer’s perspective, it feels unnatural not to be able to respond to these real-world demands “just because a study design won’t allow you to.” This challenge was also reported by two recent studies focusing on eHealth interventions in general [36] and, more specifically, in the field of psychiatry [37].

We suggest that other study designs, such as pragmatic RCTs, action research, or even real-world data, are considered to be eligible to demonstrate the effectiveness of these interventions. These designs more closely mimic a routine clinical setting from a health care provider’s perspective (ie, no double blinding or placebo-controlled setting) and allow the interventions to be altered by the supplier during the course of the study if needed. This could lower some of the existing barriers and may convince more stakeholders to participate in eHealth research.

### Conclusions

This review demonstrates that educating patients with timely medical information through their smartphones or tablets improves their levels of knowledge, medication or treatment adherence, satisfaction, and clinical outcomes, as well as having a positive effect on health care economics. These effects are most pronounced in interventions with a short duration (ie, less than a month) and with a high frequency of messaging patients (ie, once per week or more). With the knowledge that patient education is a predictor for improved outcomes and the fact that patients have obvious difficulties processing large amounts of new medical information, we suggest incorporating the delivery of timely information through smartphone and tablet apps within current medical practices.

## Appendix

Multimedia Appendix 1 MEDLINE (Medical Literature Analysis and Retrieval System Online) search strategy.

Multimedia Appendix 2 Overview of outcomes per study and instruments used to assess them.

## Abbreviations

CINAHL: Cumulative Index to Nursing and Allied Health Literature
eHealth: electronic health
MEDLINE: Medical Literature Analysis and Retrieval System Online
MeSH: Medical Subject Headings
PRISMA: Preferred Reporting Items for Systematic Reviews and Meta-Analyses
Radboudumc: Radboud University Medical Center
RCT: randomized controlled trial
SMD: standardized mean difference
