# Supplementary material for: Educating Patients by Providing Timely Information Using Smartphone and Tablet Apps: Systematic Review
Source: J Med Internet Res. 2020 Apr 13;22(4):e17342. doi: 10.2196/17342 (PMC7186866; doi:10.2196/17342)
Supplement: Multimedia Appendix 1 [file jmir_v22i4e17342_app1.pdf]

Educating patients by providing timely information using smartphone and tablet apps. A systematic review.

MEDLINE search strategy:

("Precision Medicine"[Mesh] OR "Patient Education as Topic"[Mesh])
OR adherence with instructions[tiab]
OR contextual information[tiab]
OR contextual learning[tiab]
OR individual care[tiab]
OR individual coaching[tiab]
OR individual education[tiab]
OR individual information[tiab]
OR individual instructions[tiab]
OR individual learning[tiab]
OR individual teaching[tiab]
OR individualised care[tiab]
OR individualised education[tiab]
OR individualised information[tiab]
OR individualised teaching[tiab]
OR individualized care[tiab]
OR individualized coaching[tiab]
OR individualized education[tiab]
OR individualized information[tiab]
OR individualized instructions[tiab]
OR individualized learning[tiab]
OR individualized teaching[tiab]
OR medication adherance[tiab]
OR medication compliance[tiab]
OR patient centred care[tiab]
OR patient centred education[tiab]
OR patient centred information[tiab]
OR patient centred learning[tiab]
OR patient centred teaching[tiab]
OR patient education[tiab]
OR patient information[tiab]
OR patient knowledge[tiab]
OR patient learning[tiab]
OR patient teaching[tiab]
OR perioperative care[tiab]
OR personal care[tiab]
OR personal coaching[tiab]
OR personal education[tiab]
OR personal information[tiab]
OR personal instruction[tiab]
OR personal notification[tiab]
OR personalised care[tiab]

OR personalised information[tiab]
OR personalized care[tiab]
OR personalized coaching[tiab]
OR personalized education[tiab]
OR personalized information[tiab]
OR personalized instruction[tiab]
OR personalized learning[tiab]
OR postoperative care[tiab]
OR preoperative care[tiab]
OR tailored advice[tiab]
OR tailored care[tiab]
OR tailored content[tiab]
OR tailored education[tiab]
OR tailored information[tiab]
OR tailored message[tiab]
OR tailored to the context[tiab]
OR tailored to the patient[tiab]
OR timely education[tiab]
OR timely information[tiab]
OR therapy compliance[tiab]
OR treatment compliance[tiab]
OR treatment adherence[tiab]
OR treatment preparation[Tiab]
)

AND

(
("Computers, Handheld"[Mesh] OR "Smartphone"[Mesh])
OR App[Tiab] OR Apps[Tiab]
OR cell phone[Tiab]
OR cell phones[Tiab]
OR ehealth[Tiab]
OR e-health[Tiab]
OR e-healthcare[Tiab]
OR electronic reminder application*[tiab]
OR Handheld Computer[tiab]
OR Handheld Computers[tiab]
OR mhealth[Tiab]
OR m-health[Tiab]
OR Mobile Application[Tiab]
OR Mobile Applications[Tiab]
OR Mobile App[Tiab]
OR Mobile Apps[Tiab]
OR mobile health[Tiab]
OR mobile phone[Tiab]
OR mobile phones[Tiab]

OR Mobile-health[tiab]
OR Mobile-healthcare[tiab]
OR Palm Pilot[tiab]
OR Palm Pilots[tiab]
OR Palmtop Computer[tiab]
OR Palm-Top Computer[tiab]
OR Palmtop Computers[tiab]
OR Palm-Top Computers[tiab]
OR PDA Computer[tiab]
OR PDA Computers[tiab]
OR Personal Digital Assistant[tiab]
OR Pocket PC[tiab]
OR Pocket PCs[tiab]
OR Smartphone[Tiab]
OR Smartphones[Tiab]
OR Tablet[Tiab]
OR Tablets[Tiab]
OR Push-notification[Tiab]
OR Push notification[Tiab]
OR Telemedicine[tiab]
OR Tele-medicine[tiab]
)

AND

(
"2014/01/01"[Date - Publication] : "2019/12/31"[Date - Publication]
)
