# Supplementary material for: Educating Patients by Providing Timely Information Using Smartphone and Tablet Apps: Systematic Review
Source: J Med Internet Res. 2020 Apr 13;22(4):e17342. doi: 10.2196/17342 (PMC7186866; doi:10.2196/17342)
Supplement: Multimedia Appendix 2 [file jmir_v22i4e17342_app2.pdf]

Educating patients by providing timely information using smartphone and tablet apps. A systematic review.

Overview of outcomes per study and instruments to assess them

Study	Year	Population (n)	Outcomes and instruments
Wang	2019	Colonoscopy (392)	Quality of preparation: Boston Bowel Preparation Score (P) (CR)<br>Adenoma detection: EHR (CR)<br>Satisfaction: Self-developed questionnaire (PR)
Timmers	2019	Knee replacement (212)	Pain: Pain at rest / during activity / at night NRS 0-10 (P) (PR)<br>QoL: EuroQol EQ-5D-3L (PR)<br>Physical functioning: Knee injury and Osteoarthritis Outcome Score Short Form (KOOS PS) (PR)<br>Satisfaction: Self-developed questionnaire (PR)<br>Health care consumption: Self-developed questionnaire (PR)
Mata	2019	Colorectal surgery (97)	Adherence: Assessment of Early Recovery Protocol (P) (PR)<br>Length of stay: EHR (CR)<br>Complications: EHR (CR)<br>Satisfaction: 4 items from the Consumer Assessment of Healthcare Providers and Systems Surgical Care Survey (S-CAHPS) (PR)
Li	2019	Pediatric day-care surgery (127)	Quality of recovery: Health Index (Physiological function, ability to perform daily activities, injury response) (P) (PR)<br>Satisfaction: Self-developed questionnaire (PR)<br>Time consumption during follow-up: EHR (CR)
Jeon	2019	Colonoscopy (281)	Quality of preparation: Ottawa Bowel Preparation Scale (P) (CR)<br>Adenoma Detection Rate: EHR (CR)
Van der Meij	2018	Abdominal surgery (344)	First return to normal activity: Dutch–Flemish Patient-Reported Outcomes Measurement Information System (PROMIS) Physical Function (PROMIS-PF) (P) (PR)<br>Return to work / physical activities: International Physical Activity Questionnaire (IPAQ) (PR)<br>QoL: EuroQol EQ-5D-3L (PR)<br>Pain: Pain intensity VAS 0-100 (PR)<br>Satisfaction: Self-developed questionnaire (PR)<br>Healthcare costs: EHR (CR)
Timmers	2018	Knee replacement (213)	Knowledge: Self-developed questionnaire (P) (PR)<br>Mobile device proficiency: Mobile Device Proficiency Questionnaire 16 (PR)<br>Treatment chosen: Self-developed questionnaire (PR)<br>Satisfaction: Self-developed questionnaire (PR)
Najafi Ghezeljeh	2018	Hypertension (100)	Hypertension self-management: Hypertension Self-Management Behavior Questionnaire (HSMBQ) (P) (PR)
Hardt	2018	Knee replacement (60)	Range of motion: EHR (P) (CR)<br>Pain: Pain at rest / during activity NRS 0-10 (PR)<br>Physical functioning: Knee injury and Osteoarthritis Outcome Score (KOOS) (PR) Knee Society Score (KSS) (PR)<br>Clinical outcomes: EHR (CR)
Alanzi	2018	Diabetes Mellitus (92)	Knowledge: Diabetic Knowledge Test (DKT-24) (P) (PR)<br>Self-efficacy: Diabetes Mellitus Self-Efficacy Scale (PR)
Widmer	2017	Cardiac rehabilitation (80)	In-person hospital visits: EHR (P) (CR)<br>Clinical parameters: EHR (CR)<br>QoL: Dartmouth QOL (PR)

Asklund	2017	Stress urinary incontinence (123)	Symptom severity: International Consultation on Incontinence Modular Questionnaire Urinary Incontinence Short Form [ICIQ-UI SF] (P) (PR)<br>QoL: ICIQ Lower Urinary Tract Symptoms Quality of Life [ICIQ-LUTSqol] (PR)
Sharara	2017	Colonoscopy (160)	Quality of preparation: Aronchick Scale (P), Ottawa Bowel Preparation Scale, Chicago Bowel Preparation Scale (CR)<br>Adherence to prescribed diet: Self-developed questionnaire (PR)<br>Satisfaction: Self-developed questionnaire (PR)
Perry	2017	Asthma (34)	Asthma control: Asthma Control Test (ACT) (P) (PR)<br>Expiratory volume: EHR (CR)
Lee	2017	Breast cancer (120)	Mammogram receipt: Self-developed questionnaire (P) (PR)<br>Knowledge: Breast Cancer Knowledge Test (PR)<br>Satisfaction: Self-developed questionnaire (PR)
Lakshminaraynana	2017	Parkinson's' disease (158)	Medication adherence: Morisky Medication Adherence Scale (MMAS-8) (P) (PR)<br>QoL: Parkinson's Disease Questionnaire (PDQ-39) (PR)<br>Quality of consultation: Patient-Centered Questionnaire (PCQ-PD) (PR)<br>Anxiety and depression: Hospital Anxiety and Depression Scale (HADS) (PR)<br>Beliefs about medication: Beliefs about Medication Questionnaire (PR)<br>Non-Motor symptom questionnaire: Non-Motor Symptom Questionnaire (PR)
Guo	2017	Atrial fibrillation (209)	Knowledge: Knowledge: Self-developed questionnaire (P) (PR)<br>QoL: EuroQol EQ-5D-5L (PR)<br>Adherence: Pharmacy Quality Adherence Measures (PR)<br>Satisfaction: Self-developed questionnaire (PR)
Van Reijnen	2017	Ankle trauma (220)	Incidence of ankle sprains: Self-developed questionnaire (P) (PR)<br>Residual pain and ankle disability: Functional Disability Ankle Index (FADI) (PR)
Kang	2016	Colonoscopy (650)	Bowel preparation: Ottawa Score (P) (CR)<br>Compliance with instructions: EHR (CR)<br>Cecal intubation rate: EHR (CR)<br>Adenoma Detection Rate: EHR (CR)
Johnston	2016	Myocardial infarction (174)	Medication adherence: Medication Adherence Rating Scale (MARS-5) (P) (PR)<br>Clinical outcomes: EHR (CR)<br>QoL: EuroQol EQ-5D-3L (PR)<br>Satisfaction: System Usability Score (PR)
Lorenzo-Zuniga	2015	Colonoscopy (260)	Bowel preparation: Harefield Cleansing Scale (HCS) (P) (CR)<br>Satisfaction: Self-developed questionnaire (PR)

(P): Primary outcome
QoL: Quality of Life
EHR: from the Electronic Health Record (or hospital information system)

PR: Patient Reported
CR: Clinician Reported
